# Supplementary material for: Morphometric assessment of the femoral trochlea, patella and patellar ligament in small breed dogs with and without grade II medial patellar luxation
Source: BMC Vet Res. 2026 Aug 19;22:478. doi: 10.1186/s12917-026-05816-x (PMC13488211; doi:10.1186/s12917-026-05816-x)
Supplement: Supplementary file 1 [file 12917_2026_5816_MOESM1_ESM.docx]

**ORGINAL RESEARCH ARTICLE**

**Morphometric assessment of the femoral trochlea, patella and patellar ligament in small breed dogs with and without grade II medial patellar luxation**

**Rudi Barthmann^1^, Lars FH Theyse^1^
^1^ Department for Small Animals University of Leipzig, Leipzig, Germany
Address for correspondence: Dr. med. vet. Rudi Barthmann, Department for Small Animals – University of Leipzig, Leipzig, Germany (e-mail:** [rudi.barthmann@kleintierklinik.uni-leipzig.de](mailto:rudi.barthmann@kleintierklinik.uni-leipzig.de)**);**

**Morphometric assessment of the femoral trochlea, patella and patellar ligament in small breed dogs with and without grade II medial patellar luxation**

**Abstract**

**Background:** Medial patellar luxation is a common orthopedic condition in small breed dogs, though its etiology remains poorly understood. The aim of this study was to assess the morphometry of the femoral trochlea, patella and patellar ligament in small breed dogs with and without grade 2 medial patellar luxation. This retrospective study evaluated small breed dogs weighing ≤15 kg including 80 stifles with Grade 2 MPL and 80 controls. The femoral trochlear length to patellar length ratio (TL:PL), trochlear curvature to condylar curvature ratio (TC:CC) and patellar ligament length to patellar length ratio (PLL:PL) were assessed. In addition, these parameters were determined comparing dogs with and without a chondrodysplastic phenotype. T-tests and ANOVA were used for statistical analysis.

**Results:** The TL:PL ratio was increased in dogs with grade II MPL compared to controls with 1.7 ± 0.3 and 1.6 ± 0.2. (p<.001), respectively. This indicates an increased length of the trochlea. No differences were found in the TC:CC and PLL:PL ratios between both groups. Within the grade II MPL group, there was an increased PLL:PL ratio (2.0 ± 0.2; 1.9 ± 0.2; p=.002) in dogs with chondrodysplastic phenotype in comparison with non-chondrodysplastic phenotype. A decreased femoral trochlear length, or a decreased curvature of the femoral trochlea does not seem to be related to grade II patella luxation in small breed dogs. In dogs with a chondrodysplastic phenotype the relatively longer patellar ligament could play a role in the development of grade II MPL.

**Conclusion:** Trochlear length and curvature did not seem to be related to the development of grade II MPL. I*n* dogs with a chondrodysplastic phenotype, grade II MPL could be related to a longer patellar ligament.
***Keywords:*** *medial patella luxation, morphometric measurements, stifle radiography, chondrodysplasia*

**Background**

Medial patellar luxation (MPL) is the most frequent orthopedic diseases in small breed dogs [1-3]. The disorder was shown to have a prevalence between 1,3% to 9,2% [4, 5]. MPL leads to lameness, pain and eventually osteoarthritis. Physiologically the patella lies in the trochlear groove and is mainly stabilized passively by the trochlear ridges, parapatellar cartilage, femoropatellar ligaments and retinacula. Dynamic stabilization is provided by the quadriceps femoris muscle group [6]. Patellar luxation is characterized by the direction of luxation: medial, lateral or bidirectional [7-11]. In small breed dogs, patellar luxation is more frequent than in large dogs, with medial patellar luxation (MPL) as the dominant type [2, 3]. Bilateral occurrence of MPL is found in up to 49% of affected dogs [2, 5]. Pomeranian, Chihuahua, Yorkshire Terrier, Poodle and French Bulldog are clearly overrepresented, highlighting the hereditary origin of patella luxation [4, 12, 13]. In contrast to human medicine, traumatic patellar luxation rarely occurs in dogs [2, 3, 14]. According to the modified grading system by Roush, patella luxation can be classified in four different grades, regardless of the direction [9, 15]. Grade II MPL has the highest prevalence in small breed dogs and is clinically most relevant [2,4]. The clinical signs typically are intermittent non-weightbearing lameness of the hindlimb. The exact pathogenesis of MPL still needs to be elucidated. Several developmental musculoskeletal abnormalities have been proposed to cause MPL [8, 16]. In grade II MPL, the anatomical conformations of the femoropatellar joint are mostly retained. In grade III and grade IV patella luxation the loss of physiological pressure of the patella on the trochlea results in a shallow trochlea. This is also known as trochlear dysplasia or trochlear hypoplasia [11, 17, 18]. In the end stage, the depth of the patella can be completely lost.
Another important factor is the proximodistal position of the patella in the femoral trochlea. Consistent with patella luxation in humans, a proximal position of the patella, defined as patella alta, could predispose to patellar luxation in dogs [19, 20]. In view of this, the patellar ligament length to patella length (PLL:PL) ratio was introduced for dogs similar to the Insall-Salvati ratio in humans [21-24].
In literature, MPL in medium to giant breed dogs has been associated with patella alta and an increased patellar ligament length [22, 25]. However, in small breed dogs the PLL:PL ratio was not related to MPL [10, 16, 23, 24, 26-28]. To assess the relative proximodistal position of the patella, the relation between the insertion of the patellar ligament on the tibial tuberosity and the tibial plateau was determined. Small breed dogs with MPL showed a more proximal position of the patellar ligament insertion and patella [23]. A more proximal position of the patella could also be related to a reduced length of the femoral trochlea. Proximal lengthening of the femoral trochlea has been used successfully in the management in MPL [29]. In addition, trochlear configuration seems to play a role in patellofemoral contact mechanics, influencing both contact area and pressure distribution within the canine stifle joint[30, 31]. Therefore, an altered curvature of the femoral trochlea could play a role in a reduced patellar-trochlear contact and patellar instability. Grade II MPL is generally associated with mild or inconsistent changes in trochlear depth, in contrast to the more pronounced trochlear hypoplasia observed in advanced grades [32].
Grade III and grade IV MPL preclude radiographic measurement in a mediolateral view due to distortion of ligaments and bone structures. In grade II MPL, the patella is typically positioned in the femoral trochlea during radiographic examination.
At present, studies concerning the influence of a chondrodysplastic phenotype on the development of MPL is lacking. Dogs with a chondrodysplastic phenotype (CD) are characterized by decreased bone length development resulting in alteration of the bone and soft tissue relation in comparison with non-chondrodysplastic (NCD) dogs. Patella length, patellar ligament length, femoral trochlear length, trochlear curvature could be different in dogs with a CD phenotype.
The aim of this study was to evaluate morphometric differences assessing trochlear length and curvature, the patellar ligament length and patellar length in small breed dogs with grade II MPL. In addition, we investigated potential differences of these parameters between chondrodysplastic and non-chondrodysplastic phenotypes.

**Methods**

*Study design*

Medical records of dogs admitted to the teaching hospital from 2016 to 2022 were retrospectively reviewed. Small breed dogs with a body weight ≤15 kg diagnosed with Grade II MPL, a documented orthopedic examination, and a complete set of stifle radiographs of at least one stifle were included. A 4-grade clinical classification system was used as previously described (Table 1). [15] The control group consisted of small breed dogs with a body weight ≤15 kg without patella luxation and similar weight and size distribution. The femoral trochlear length to patellar length ratio (TL:PL), the trochlear to condylar curvature ratio (TC:CC) and the patellar ligament length to patellar length ratio (PLL:PL) were determined. Measurements were performed on mediolateral radiographs of the stifle, as similar previously described measurements. [22-25] Normal standing angles of the stifle joint, ranging from 125° to 155°, were applied. Accurate positioning relied on superimposition of the femoral condyles. The trochlear length was determined by measuring from the most proximal part of the trochlea to the junction between trochlea and femoral condyle. The patellar length was determined as the most proximal aspect of the base of the patella to the most distal aspect of the patellar apex. Patellar ligament length was determined from the distal end of the patellar apex to the most distal point of insertion of the patellar ligament on the tibial tuberosity (Figure 1). [25] Best-fit circles were superimposed to the osseous contours of the femoral trochlear groove and femoral condyles as previously described [33, 34]. The ratio of the best-fit trochlear (TC) and condylar circles (CC) and its surface area was used as a dimensionless measure (TC:CC) of the femoral trochlear curvature. (Figure 2). The measurements were exclusively done by the first author avoiding interobserver error. Ratios were used for all assessments, leading to independency of dog size or magnification of radiographic images. All radiographs were examined with blinding of groups. An imaging software was used for radiographic measurements (IntelliSpace PACS, Philips, Eindhoven, Netherlands). After performing all prior measurements, dogs were also assessed based on their CD or NCD phenotype.

*Statistical analysis*

Descriptive statistical analysis and the Shapiro-Wilk test were used to confirm normal distribution for both groups and for all 4 groups after separation in CD and NCD. T-tests were applied to compare the means of the TL:PL, TC:CC and the PLL:PL ratio between the grade II MPL group and the control group. After separation in CD and NCD phenotype, ANOVA tests were applied to test the variances of the group means of all four groups for the three ratios. Post hoc analysis with Fishers LSD test was used for significant ANOVA tests. Statistical significance was considered for p-values <.05. Data analysis was conducted using the statistical package of MS Excel (Microsoft Excel, Microsoft, Redmond, Washington, USA) and IBM SPSS statistics version 27 (IBM, Armonk, New York, USA).  **Results:**

The study included a total of 124 dogs. The grade II MPL group consisted of 54 dogs with 80 stifle radiographs in total. The group without MPL included 80 stifles radiographs. The population showed several breeds and cross breeds with an overrepresentation of Chihuahuas, French Bulldogs and Yorkshire Terriers. The distribution of male and female and neutered and intact dogs was similar between groups. The mean weight ± SD for the grade II MPL group was 7.6 ± 3.3 kg, for the group without MPL 9.8 ± 3.3 kg. The mean age ± SD for the grade II MPL group was 4.4 ± 3.2 years and for the group without MPL 7.1 ± 3.6 years. The grade II MPL group consisted of 40 stifle radiographs of dogs with a CD phenotype and 41 of dogs with a NCD phenotype. In the group without MPL the numbers were 30 and 54, respectively. The TL:PL ratio was significantly higher in dogs with grade II MPL than in controls (p<.001; 1.74 ± 0.31 and 1.60 ± 0.16, respectively). There were no differences in the TC:CC ratios and PLL:PL ratios between groups. However, dogs with grade II MPL and a CD phenotype showed an increase of the PLL:PL ratio in comparison with grade II MPL dogs with an NCD phenotype (1,97 ± 0.20; 1.93 ± 0.21; p=.002, respectively). ~~meaning an increased patellar ligament length~~. In addition, there was a difference in PLL:PL ratios between the grade II MPL and control group (1,93 ± 0,21; 1,98 ± 0,16; p=.01, respectively) within the dogs with a NCD phenotype.~~, meaning a shorter patellar ligament~~. Comparison of the TL:PL and the TC:CC ratios for the MPL and controls within CD and NCD phenotypes, showed no differences. Irrespective of a CD and NCD phenotype, grade II MPL TL:PL ratios remained significantly higher in comparison with controls (Table 2 and Table 3 and Table 4).

**Discussion:**

Our study evaluated morphometric differences assessing trochlear length and curvature, the patellar ligament length and patellar length in small breed dogs with and without grade II MPL. We found a higher TL:PL ratio in dogs with grade II MPL. Although most literature uses the patellar length as reference, alterations in the PL would influence the TL:PL and PLL:PL ratio. This longer femoral trochlea would suggest that hypothesized patella alta does not seem to play a role in the development of grade II MPL. In contrast, the longer femoral trochlea length could be an adaptation in dogs with functional patella alta. This concept was confirmed by S Murakami, M Shimada and Y Hara [35] describing patella alta during full extension of the stifle. In view of this, preventing hyperextension of the stifle joint might be important in the management of medial patella luxation as the increase in femoral trochlear length, found in our study, was insufficient to prevent MPL.
Interestingly, this increased femoral trochlear length is in contrast to previous findings by S Murakami, M Shimada, Y Harada and Y Hara [36] who found a decreased femoral trochlea length in affected dogs. This discrepancy may be explained by differences in breed and phenotype distribution or by the inclusion of dogs with grade I–III MPL in their study, whereas only dogs with grade II MPL were included in the present study. Our study population consisted of a larger portion of French bulldogs with a chondrodysplastic phenotype. To evaluate the possible influence of a CD phenotype on the morphometric measurements, we assigned the dogs accordingly.
We found no differences in the PLL:PL ratio in dogs with and without MPL. This means that there is no direct indication for a more proximal position of the patella in the femoral trochlea, which is consistent with previous findings in small breed dogs [23, 24, 27, 37]. Interestingly, in dogs with a CD phenotype and grade II MPL the PLL:PL ratio was significantly higher, which would be consistent with a longer patellar ligament length. Dogs with a CD phenotype have decreased length of long bones, but a less pronounced shorting of soft tissues and ligaments. This might explain the relative increase of the PLL:PL ratio with a longer patellar ligament. It is unclear, whether dogs with a CD phenotype could have an altered patellar length. In contrast, the decreased PLL:PL ratio in dogs with grade II MPL and an NCD phenotype would imply a shorter patellar ligament. There is no description of elongation of the patella in NCD phenotype dogs. The decreased PLL:PL ratio contradicts a patella alta as causative factor. However, dynamic hyperextension of the stifle joint could predispose for MPL. To the authors knowledge, the assessment of the curvature of the femoral trochlea and femoral condyle defined as the TC:CC ratio to assess the configuration of the trochlea has not been described before. Nevertheless, we did not find any differences in these ratios. This would imply that the configuration of the femoral trochlea does not play a role in the development grade II MPL. The following limitations of our study must be considered. The present study has a relatively small sample size, particularly after dividing the dogs according to phenotype. Consequently, subgroups contained only a limited number of dogs, which may have reduced statistical power.
Although superimposition of the femoral condyle was used to standardize the position of the stifle joint, slight variations were accepted. This could lead to suboptimal measurements. An additional limitation was, that measurements assessed the greatest trochlear length. However, the trochlear ridges usually are different in length on the medial and lateral side. Although morphometric assessment was done by one person repeatedly, measurement inconsistencies cannot be excluded. Another measurement limitation is the evaluation of a three-dimensional structures in only two dimensions. Three-D reconstruction computed tomography could theoretically improve accuracy. In using ratios instead of absolute measurements, we minimized the dependency on the patient size and magnification of the radiographic image.[38] As phenotype seems to influence morphometric measurements, assessment within breeds could lead to more specific insights in the development of MPL.

**Conclusion:**

An increased length of the femoral trochlea in grade II MPL affected dogs could potentially be a result of a compensatory mechanism, nevertheless failing to prevent the development of MPL. The femoral trochlea length and curvature did not seem to be related to grade II **MPL. In dogs with a CD phenotype, a relative longer patellar ligament could be a factor in the development of** grade II **MPL.**

**References:**

1.	Ramírez-Flores GI, Del Angel-Caraza J, Quijano-Hernández IA, Hulse DA, Beale BS, Victoria-Mora JM: **Correlation between osteoarthritic changes in the stifle joint in dogs and the results of orthopedic, radiographic, ultrasonographic and arthroscopic examinations**. *Veterinary Research Communications* 2017, **41**(2):129-137.

2.	Alam MR, Lee JI, Kang HS, Kim IS, Park SY, Lee KC, Kim NS: **Frequency and distribution of patellar luxation in dogs**. *Vet Comp Orthop Traumatol* 2007, **20**(01):59-64.

3.	Engdahl K, Hanson J, Bergström A, Bonnett B, Höglund O, Emanuelson U: **The epidemiology of stifle joint disease in an insured Swedish dog population**. *Veterinary Record* 2021, **189**(3):e197.

4.	O’Neill DG, Meeson RL, Sheridan A, Church DB, Brodbelt DC: **The epidemiology of patellar luxation in dogs attending primary-care veterinary practices in England**. *Canine Genetics and Epidemiology* 2016, **3**(1):4.

5.	Bosio F, Bufalari A, Peirone B, Petazzoni M, Vezzoni A: **Prevalence, treatment and outcome of patellar luxation in dogs in Italy**. *Vet Comp Orthop Traumatol* 2017, **30**(05):364-370.

6.	DeCamp CE: **Brinker, Piermattei and Flo's handbook of small animal orthopedics and fracture repair**: Elsevier Health Sciences; 2015.

7.	Harasen G: **Patellar luxation: pathogenesis and surgical correction**. *Can Vet J* 2006, **47**(10):1037-1039.

8.	Hulse DA: **Pathophysiology and management of medial patellar luxation in the dog**. *Veterinary medicine, small animal clinician : VM, SAC* 1981, **76**(1):43-51.

9.	Perry KL, Déjardin LM: **Canine medial patellar luxation**. *Journal of Small Animal Practice* 2021, **62**(5):315-335.

10.	Wangdee C, Hazewinkel HAW, Temwichitr J, Theyse LFH: **Extended proximal trochleoplasty for the correction of bidirectional patellar luxation in seven Pomeranian dogs**. *Journal of Small Animal Practice* 2015, **56**(2):130-133.

11.	Di Dona F, Della Valle G, Fatone G: **Patellar luxation in dogs**. *Vet Med (Auckl)* 2018, **9**:23-32.

12.	Wangdee C, Leegwater PA, Heuven HC, van Steenbeek FG, Techakumphu M, Hazewinkel HA: **Population genetic analysis and genome-wide association study of patellar luxation in a Thai population of Pomeranian dogs**. *Res Vet Sci* 2017, **111**:9-13.

13.	Nganvongpanit K, Yano T: **Prevalence of and Risk Factors of Patellar Luxation in Dogs in Chiang Mai, Thailand, during the Years 2006-2011**. *Thai Journal of Veterinary Medicine* 2011, **41**(4):449-454.

14.	Sillanpää P, Mattila VM, Iivonen T, Visuri T, Pihlajamäki H: **Incidence and risk factors of acute traumatic primary patellar dislocation**. *Medicine and science in sports and exercise* 2008, **40**(4):606-611.

15.	Roush JK: **Canine patellar luxation**. *Vet Clin North Am Small Anim Pract* 1993, **23**(4):855-868.

16.	Towle HA, Griffon DJ, Thomas MW, Siegel AM, Dunning D, Johnson A: **Pre- and Postoperative Radiographic and Computed Tomographic Evaluation of Dogs with Medial Patellar Luxation**. *Veterinary Surgery* 2005, **34**(3):265-272.

17.	Petazzoni M, De Giacinto E, Troiano D, Denti F, Buiatti M: **Computed Tomographic Trochlear Depth Measurement in Normal Dogs**. *Vet Comp Orthop Traumatol* 2018, **31**(6):431-437.

18.	Garnoeva RS: **Evaluation of Trochlear Dysplasia in Dogs with Medial Patellar Luxation - Comparative Studies**. *Acta Scientiae Veterinariae* 2021, **49**:7.

19.	Simmons E, Cameron JC: **Patella alta and recurrent dislocation of the patella**. *Clin Orthop Relat Res* 1992, **274**:265-269.

20.	Magnussen RA, De Simone V, Lustig S, Neyret P, Flanigan DC: **Treatment of patella alta in patients with episodic patellar dislocation: a systematic review**. *KSSTA* 2014, **22**:2545-2550.

21.	Insall J, Salvati E: **Patella position in the normal knee joint**. *Radiology* 1971, **101**(1):101-104.

22.	Johnson AL, Broaddus KD, Hauptman JG, Marsh S, Monsere J, Sepulveda G: **Vertical patellar position in large-breed dogs with clinically normal stifles and large-breed dogs with medial patellar luxation**. *Vet Surg* 2006, **35**(1):78-81.

23.	Feldmane L, Theyse LFH: **Proximodistal and caudocranial position of the insertion of the patellar ligament on the tibial tuberosity and patellar ligament length of normal stifles and stifles with grade II medial patellar luxation in small-breed dogs**. *Vet Surg* 2021, **50**(5):1017-1022.

24.	Wangdee C, Theyse LFH, Hazewinkel HAW: **Proximo-distal patellar position in three small dog breeds with medial patellar luxation**. *Veterinary and Comparative Orthopaedics and Traumatology* 2015, **28**(4):270-273.

25.	Mostafa AA, Griffon DJ, Thomas MW, Constable PD: **Proximodistal alignment of the canine patella: radiographic evaluation and association with medial and lateral patellar luxation**. *Vet Surg* 2008, **37**(3):201-211.

26.	Mortari AC, Rahal SC, Vulcano LC, da Silva VC, Volpi RS: **Use of radiographic measurements in the evaluation of dogs with medial patellar luxation**. *Can Vet J* 2009, **50**(10):1064-1068.

27.	Yasukawa S, Edamura K, Tanegashima K, Seki M, Teshima K, Asano K, Nakayama T, Hayashi K: **Evaluation of bone deformities of the femur, tibia, and patella in Toy Poodles with medial patellar luxation using computed tomography**. *Vet Comp Orthop Traumatol* 2016, **29**(1):29-38.

28.	Garnoeva RS: **Evaluation of proximodistal patellar alignment in small breed dogs with or without patellar luxation using the Insall-Salvati, Caton-Deschamps, and Blackburne-Peel indices**. *Open veterinary journal* 2023, **13**(5):663-667.

29.	Wangdee C, Theyse LF, Techakumphu M, Soontornvipart K, Hazewinkel HA: **Evaluation of surgical treatment of medial patellar luxation in Pomeranian dogs**. *Vet Comp Orthop Traumatol* 2013, **26**(6):435-439.

30.	Yoo YH, Lee SJ, Jeong SW: **Effects of quadriceps angle on patellofemoral contact pressure**. *Journal of veterinary science* 2020, **21**(5):e69.

31.	Park D, Kang J, Kim N, Heo S: **Patellofemoral contact mechanics after transposition of tibial tuberosity in dogs**. *Journal of veterinary science* 2020, **21**(4):e67.

32.	Morishima T, Tanegashima K, Tomo Y, Eto H, Yamazaki A, Edamura K: **An assessment of trochlear groove morphology with the severity of medial patellar luxation using ultrasonography in dogs presented to a clinic in Japan**. *Veterinary record open* 2025, **12**(1):e70010.

33.	Pilli M, Seyrek Intas D, Etikan I, Yigitgor P, Kramer M, Tellhelm B, von Puckler K: **The Role of Femoral Head Size and Femoral Head Coverage in Dogs with and without Hip Dysplasia**. *Veterinary Sciences* 2023, **10**(2):120.

34.	Nötzli HP, Wyss TF, Stoecklin CH, Schmid MR, Treiber K, Hodler J: **The contour of the femoral head-neck junction as a predictor for the risk of anterior impingement**. *The Journal of bone and joint surgery British volume* 2002, **84**(4):556-560.

35.	Murakami S, Shimada M, Hara Y: **Examination of Proximodistal Patellar Position in Dogs with the Stifle at Full Extension**. *Vet Comp Orthop Traumatol* 2023, **36**(04):199-206.

36.	Murakami S, Shimada M, Harada Y, Hara Y: **Examination of the proximodistal patellar position in small dogs in relation to anatomical features of the distal femur and medial patellar luxation**. *Plos One* 2021, **16**(5).

37.	Tomo Y, Edamura K, Yamazaki A, Tanegashima K, Seki M, Asano K, Tinga S, Hayashi K: **Evaluation of Hindlimb Deformity and Posture in Dogs with Grade 2 Medial Patellar Luxation during Awake Computed Tomography Imaging while Standing.** *Vet Comp Orthop Traumatol.* 2022 May;**35**(3):143-151. doi: 10.1055/s-0041-1740607.

38.	Johnson AL, Probst CW, DeCamp CE, Rosenstein DS, Hauptman JG, Kern TL: **Vertical position of the patella in the stifle joint of clinically normal large-breed dogs**. *Am J Vet Res* 2002, **63**(1):42-46.

**Tables:**

Table 1: Patella luxation clinical grading system

| Grade | Description |
| --- | --- |
| I | The patella can be manually luxated with little force but promptly returns to a normal position after releasing the force |
| II | The patella may luxate during motion of the stifle or by manual force and remains luxated until stifle extension or manual replacement occurs |
| III | The patella is permanently luxated but can be manually reduced. It reluxates spontaneously when manual force is removed. |
| IV | The patella is permanently luxated and cannot be manually reduced |

Table 2: Mean, standard deviation and p-value of the ratios TL:PL, PLL:PL, TC:CC in grade II MPL dogs and controls. TL – trochlear length, PL – patella length, PLL – patellar ligament length, TC – trochlear best-fit circle area, CC – best-fit condylar circle area

|  | TL:PL | PLL:PL | TC:CC |
| --- | --- | --- | --- |
| MPL-group | 1.74 ± 0.31 | 1,98 ± 0,2 | 2,38 ± 0,68 |
| Control group | 1.60 ± 0.16 | 1,97 ± 0,18 | 2,43 ± 0,63 |
| p value | *p<.001* | p=.727 | p=.7 |

Table 3: Mean, standard deviation and p-value for dogs within the MPL or control groups separated in a chondrodysplastic (CD) or non-chondrodysplastic (NCD) phenotype. The table shows a comparison within the MPL and control group. TL – trochlear length, PL – patella length, PLL – patellar ligament length, TC – trochlear best-fit circle area, CC – best-fit condylar circle area. n.a. – not applicable as non-significant in the ANOVA. * significant in the post hoc Fishers LSD analysis.

|  |  | TL:PL | PLL:PL | TC:CC |
| --- | --- | --- | --- | --- |
| MPL-group | CD | 1,74 ± 0,21 | 1,97 ± 0,20 | 2,43 ± 0,64 |
|  | NCD | 1,65 ± 0,18 | 1,93 ± 0,21 | 2,48 ± 0,63 |
| P value (LSD) |  | n.a. | *p= .002** | n.a. |
| Control group | CD | 1,54 ± 0,16 | 1,99 ± 0,19 | 2,44 ± 0,72 |
|  | NCD | 1,57± 0,14 | 1,98 ± 0,16 | 2,42 ± 0,54 |
| p value (LSD) |  | n.a. | n.a. | n.a. |

Table 4: Mean, standard deviation and p-value for dogs within the chondrodysplastic (CD) or non-chondrodysplastic (NCD) phenotypes separated in the MPL or control group. TL – trochlear length, PL – patella length, PLL – patellar ligament length, TC – trochlear best-fit circle area, CC – best-fit condylar circle area. n.a. – not applicable as non-significant in the ANOVA. * significant in the post hoc Fishers LSD analysis.

|  |  | TL:PL | PLL:PL | TC:CC |
| --- | --- | --- | --- | --- |
| CD | MPL-group | 1,74 ± 0,21 | 1,97 ± 0,20 | 2,43 ± 0,64 |
|  | Control group | 1,54 ± 0,16 | 1,99 ± 0,19 | 2,44 ± 0,72 |
| P value |  | *p<.001** | n.a. | n.a. |
| NCD | MPL-group | 1,65 ± 0,18 | 1,93 ± 0,21 | 2,48 ± 0,63 |
|  | Control group | 1,57± 0,14 | 1,98 ± 0,16 | 2,42 ± 0,54 |
| p value |  | *p=.026** | *p=.010** | n.a. |

**Legends:**
Figure 1: Morphometric measurements on medio-lateral radiographs, determining the patellar length (PL, marked in red), the length of the trochlea (TL) and the patellar ligament length (PLL)
Figure 2: Delineation of the circles superimposed on the femoral trochlear and femoral condyle to assess the trochlear curvature (TC) and condylar curvature (CC) as the TC:CC ratio using the surface area of both circles.

## **Declaration**

**Ethics approval and consent to participate:** This study was deemed exempt from formal ethical approval due to its retrospective design using anonymised medical records. Owner consent was not required because the study used anonymised data collected as part of the routine clinical care process.

**Consent for publication:** n.a.

**Availability of data and materials:** The datasets used and/or analysed during the current study are available from the corresponding author on reasonable request.

**Competing interests:** The authors declare that they have no competing interests

**Funding:** This research did not receive any specific grant from funding agencies in the public, commercial, or not-for-profit sectors.

**Authors' contributions CRediT:** **RB**: Conceptualization, Formal analysis, Visualization, Writing – original draft; **LT**: Conceptualization, Methodology, Project administration, Resources, Supervision, Validation, Writing – review and editing

**Acknowledgements:** Prepublished Data**:** A part of the results was presented at the DVG-Vet Congress (DVG – German Veterinary Society) at the 23. November 2023, in Berlin.
